# Highly nonlinear trion-polaritons in a monolayer semiconductor

**DOI:** 10.1038/s41467-020-17340-z

**Published:** 2020-07-17

**Authors:** R. P. A. Emmanuele, M. Sich, O. Kyriienko, V. Shahnazaryan, F. Withers, A. Catanzaro, P. M. Walker, F. A. Benimetskiy, M. S. Skolnick, A. I. Tartakovskii, I. A. Shelykh, D. N. Krizhanovskii

**Affiliations:** 1 Department of Physics and Astronomy, The University of Sheffield, Sheffield, S3 7RH UK; 2 Department of Physics and Astronomy, University of Exeter, Stocker Road, Exeter, EX4 4QL UK; 3 Department of Physics and Engineering, ITMO University, St. Petersburg, 197101 Russia; 4 Institute of Physics, Polish Academy of Sciences, Al. Lotnikow 32/46, 02-668 Warsaw, Poland; 5 College of Engineering, Mathematics and Physical Sciences, University of Exeter, Exeter, EX4 4QF UK; 6 Science Institute, University of Iceland, Dunhagi-3, IS-107 Reykjavik, Iceland

**Keywords:** Nonlinear optics, Microresonators

## Abstract

Highly nonlinear optical materials with strong effective photon-photon interactions are required for ultrafast and quantum optical signal processing circuitry. Here we report strong Kerr-like nonlinearities by employing efficient optical transitions of charged excitons (trions) observed in semiconducting transition metal dichalcogenides (TMDCs). By hybridising trions in monolayer MoSe_2_ at low electron densities with a microcavity mode, we realise trion-polaritons exhibiting significant energy shifts at small photon fluxes due to phase space filling. We find the ratio of trion- to neutral exciton–polariton interaction strength is in the range from 10 to 100 in TMDC materials and that trion-polariton nonlinearity is comparable to that in other polariton systems. The results are in good agreement with a theory accounting for the composite nature of excitons and trions and deviation of their statistics from that of ideal bosons and fermions. Our findings open a way to scalable quantum optics applications with TMDCs.

## Abstract (web summary)

Here, the authors report the creation of trion-polaritons in monolayer MoSe_2_ in an open microcavity exhibiting strong nonlinear interactions, one order of magnitude bigger than those observed for exciton polaritons in GaAs.

## Introduction

Strong optical Kerr nonlinearity can be realised in photonic systems where light is resonantly coupled with optically active medium^1^. These can correspond to optical transitions in semiconductor quantum dots^2,3^, single molecules^4^ and Rydberg atoms^5^; the scalability and integration into photonic devices, nevertheless, remains a significant challenge. These obstacles can be overcome by employing hybrid 2D exciton–photon (2D polariton) systems^6,7^, enabling large effective photon–photon interactions as well as scalability and ultrafast response^8–11^. So far nonlinear polaritons have been investigated in neutral exciton–polariton platform based on GaAs quantum wells and at low temperatures (4–70 K). Interaction-based effects such as polariton Bose–Einstein condensation and superfluidity^6^, solitons^12^, quantum emission^11,13,14^ as well as polariton transistors/switches^15,16^ have been reported.

Recently, layered materials such as graphene and transition metal dichalcogenides (TMDCs)^17^ have arisen as very promising optically active 2D media offering compatibility and ease of integration with various nanophotonic devices^18^. Optical bistability and regenerative oscillations have been demonstrated in hybrid Si-graphene microcavities^19^. In contrast to graphene, monolayers of TMDCs host Wannier–Mott excitons with a huge binding energy of about 200–500 meV and large oscillator strength^20^. This enables polariton formation in photonic structures with just a single TMDC monolayer^21^ and at room temperature^22,23^, offering a major advantage over other semiconductor polariton platforms (such as GaAs). Importantly, the strong Coulomb interactions give rise to very robust 2D trions (charged excitons) in TMDCs. The large oscillator strength of trions enables formation of well-resolved trion-polariton resonances^24^ at relatively small electron density^21,25^, which, as we show here, leads to a pronounced phase space filling effect enabling nonlinearity of one to two orders of magnitude bigger (depending on exciton–photon detuning) than that of neutral exciton–polaritons in TMDC platform. Furthermore, the nonlinear refractive index (*n*_2_) per single TMDC monolayer due to trion-polaritons is estimated to be three to five orders of magnitude greater than in bare 2D TMDC materials and graphene studied in the weak light–matter coupling regime. We also probe nonlinearities due to neutral exciton–polaritons, which are observed to decrease by more than an order of magnitude with power. Such a result is explained by three-exciton and possibly trion-mediated exciton–exciton scattering processes. Overall, our work opens a new highly nonlinear system for quantum optics applications enabling in principle scalability and control through nano-engineering of van der Waals heterostructures.

## Results

### Experimental setup

**Fig. 1:**
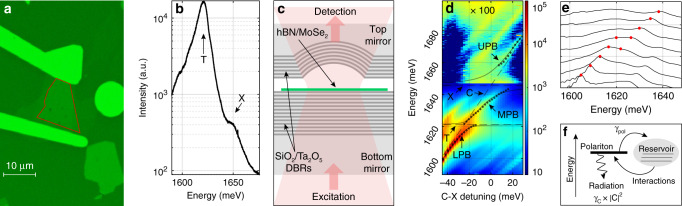
Strong light–matter coupling with MoSe_2_ monolayer. **a** Optical microscope image of the MoSe_2_ flake, highlighted with a red border, encapsulated in hBN and clamped with two golden contacts on top of the bottom mirror. **b** PL emission of the flake under off-resonant excitation with exciton (X) and trion (T) peaks highlighted. The observed order of magnitude difference for the intensity of the PL of the trion relative to the exciton arises from fast neutral exciton to trion states relaxation processes due to scattering with phonons and electrons. **c** Scheme of experiment for resonant excitation of the open cavity in the transmission geometry. **d** PL emission as a function of the detuning between bare cavity *LG*_00_ mode (C) and exciton (X) plotted in logarithmic colour scale; black points (dots) are experimental peak positions of the polariton resonances extracted from the fitting of experimental spectra at each detuning with a Gaussian; solid lines are theoretical polariton branches. Higher order transverse LG modes are also observed on the left and right-hand side of *LG*_00_ polariton states. **e** Normalised PL spectra around trion resonance, showing anticrossing behaviour. **f** Schematic showing emission and absorption of driven polariton resonance and interactions with the reservoir of trions and excitons.

In our experiment, a monolayer of MoSe_2_ (Fig. 1a) was placed into an open-access microcavity (MC) (see Fig. 1c^26^) where Laguerre–Gauss (LG) photonic modes strongly couple to exciton and trion states forming polaritons. Figure 1b shows the spectrum of PL emission from the monolayer. The strong peak at lower energy  ~1.62  eV arises from the trion (T) emission due to the natural doping in the sample, while the weaker peak at ~1.65 eV corresponds to neutral (X) excitons. To characterise the polaritons the MC was excited with a laser at 1.95 eV and polariton emission was recorded as a function of the detuning between the excitons and *LG*_00_ mode. In Fig. 1d the lower, middle and upper polariton branches (labelled LPB, MPB and UPB) are observed due to the strong coupling of the photon mode with T and X, respectively. The peak polariton positions (black dots in Fig. 1d, e) were fitted using a model of three coupled oscillators (see Supplementary Information, Notes 1 and 3, for the details).

### Nonlinear trion-polaritons

In order to probe the polariton nonlinearity polaritons were excited resonantly using a laser with 100-fs pulse duration. The excited polaritons then decay via photon emission through the DBRs with a rate ${\gamma }_{{\rm{C}}}{\left|C\right|}^{2}$ given by the cavity mode linewidth *γ*_C_ ~0.4 meV (${\left|C\right|}^{2}$ is the photonic fraction) or are absorbed in the 2D material with a rate given by the polariton linewidth *γ*_pol_ (*γ*_pol_ ~ 3–5 meV $\gg {\gamma }_{{\rm{C}}}{\left|C\right|}^{2}$) due to scattering with exciton disorder resulting in creation of an exciton-trion reservoir (Fig. 1f)^26,27^. Measurements of the intensity of transmitted light enable us to estimate the polariton density excited inside the MC and the density of the reservoir. Monitoring the frequency shift of the polariton resonance with increasing pump power, we observe the influence of interaction between polaritons and the exciton-trion reservoir (Fig. 1f), and can extract the strength of exciton- and trion-based nonlinearity.

We first present the results for interacting trion-polaritons. In this experiment the laser energy is fixed at approximately the trion level, *E*_p_ = 1.62 eV, and the transmission spectrum is recorded as the energy of the photon *LG*_00_ mode is scanned through the trion resonance. Since the linewidth of the pulsed laser (~15 meV) is significantly larger than the observed photon–trion Rabi-splitting of *ℏ*Ω_T_ ≈ 5.8 meV, it was possible to inject a similar number of polaritons in the vicinity of the trion resonance for each cavity mode position. Excitation of the UPB is negligible since it is located at energies more than ~30 meV above the trion-polariton states.

**Fig. 2:**
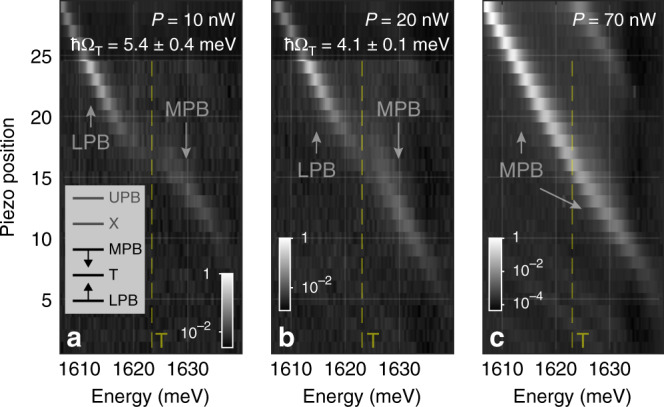
Nonlinear quench of trion-polariton splitting. **a**–**c** Cavity scan of *LG*_00_ mode across the trion resonance for different pump powers: 10, 20 and 70 nW, respectively. The peak energy of the pump laser is fixed at  ~1.62 eV. Pseudo-colour scale is logarithmic. Estimates for the trion Rabi splittings are obtained by fitting extracted peak positions for each piezo step with a coupled oscillator model. Inset: schematic diagram showing how the energies MPB and LPB are renormalised in the case of phase space filling effect leading to reduction of Rabi-splitting between trion level and the bare photon mode.

**Fig. 3:**
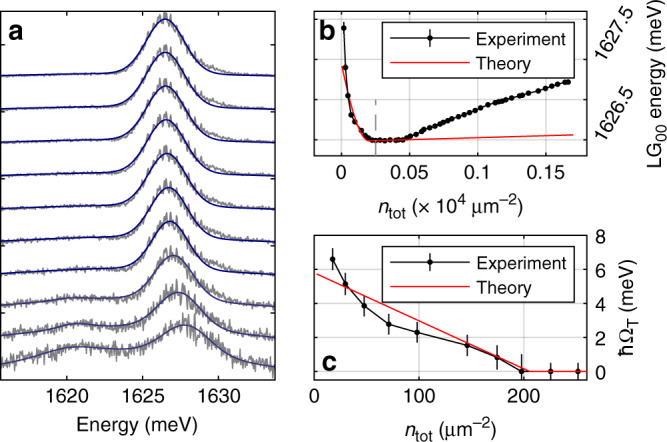
Trion-polariton nonlinearity. We consider large cavity–exciton detuning *δ*_C–X_ = −15.4 meV. **a** Spectra of the MPB and LPB for the excitation power from 5 to 50 nW (power increases from bottom to top). The MPB and LPB peaks are fitted with Gaussians. The fitting of the exciton-like LPB peak (lower energy peak) is performed for the first 4 powers, since it becomes broad and weak at higher densities and the fitting procedure is not reliable. **b** Extracted peak positions of the MPB mode vs estimated total exciton-trion density. **c** Trion-polariton Rabi-splitting vs exciton-trion density. Red solid curves in **b**, **c** correspond to the theoretical modelling results. The error bars [95% confidence interval (CI)] are estimated taking into account the random error in determination of the peak positions in **a** as well as possible systematic error (ΔΩ_T_ ~ 0.5 meV) due to the uncertainty of the fitting parameters in the coupled oscillators model.

Figure 2 shows the results of three cavity scans performed for different pump powers. At the lowest pulse power, 10 nW (Fig. 2a), an anti-crossing between the cavity mode and the trion level is clearly observed, as in the case under the non-resonant excitation. With an increase of the pump power the photon–trion Rabi-splitting is reduced (Fig. 2b) leading to the blueshift and redshift of the LPB and MPB states in the vicinity of trion resonance (Fig. 2a, inset), respectively. At *P* = 70 nW no anticrossing is observed (Fig. 2c), with MPB and LPB merging together and forming a single polariton branch. To quantify the strength of the nonlinearity, we recorded the spectra of the transmitted light as a function of pump power at a fixed photon mode energy near the trion resonance (Fig. 3a), at a fixed photon–exciton detuning *δ*_C–X_ = −15.4 meV. In Fig. 3a two peaks are observed at low power of 5 nW; the MPB peak has a higher intensity due to the higher photonic fraction. With increase of the pulse power, the MPB peak exhibits a fast redshift. The intensity of the trion-like LPB quickly reduces down to zero with power due to reduction of its photon fraction as the strong trion–photon coupling collapses.

The collapse of the strong coupling is driven by the population of both polaritons inside the cavity and reservoir determining the total density of excited excitons and trions *n*_tot_ (see Supplementary Note 1.2, for estimation of *n*_tot_). The reservoir may consist of localised or dark exciton/trion states as well as high-momenta trions degenerate with trion-polariton resonances^26,27^. For the case of resonantly driven trion-polaritons *n*_tot_ is dominated mostly by the trion density *n*_T_ (*n*_tot_ ≈ *n*_T_), since the peak of the exciton density of states is blue detuned by ~30 meV from the trion.

Figure 3b shows that the MPB energy shifts rapidly to the red wavelengths with *n*_tot_ at densities *n*_tot_ < 2 × 10^2^ μm^−2^ and then exhibits a plateau corresponding to the quenching of trion–photon coupling, followed by a gradual increase. From the redshift of the MPB branch in Fig. 3b we plot (see Supplementary Note 1.4) the value of the Rabi-splitting *ℏ*Ω_T_ vs *n*_tot_ in Fig. 3c. The collapse of trion–photon coupling occurs at a small density of *n*_tot_ ~ 2 × 10^2^  μm^−2^ very close to half the estimated density of free electrons *n*_e_/2 ≈ 2 × 10^2^ μm^−2^ (see Supplementary Note 3.2). The effective strength of the trion-polariton nonlinearity ${\beta }_{{\rm{T}}}^{{\rm{eff}}}$ responsible for the quenching of strong coupling (and large energy shifts) is defined as ${\beta }_{{\rm{T}}}^{{\rm{eff}}}=-d(\hslash {\Omega }_{{\rm{T}}})/d{n}_{{\rm{tot}}}$^28^. From Fig. 3c we deduce an average value of ${\beta }_{{\rm{T}}}^{{\rm{eff}}}\simeq 37\pm 3\,\upmu {\rm{eV}}\cdot \upmu {{\rm{m}}}^{2}$ (see Supplementary Note 1.4). Note, that the 3 μeV  ⋅ μm^2^ error above is a random error arising from the uncertainty in *ℏ*Ω_T_ in Fig. 3c and does not account for possible systematic error of *n*_tot_, which is discussed below and in Supplementary Information.

To explain the observed result we account for phase space filling effects which become important at increasing excited trion density (see Supplementary Note 3). This leads to the quenching of the collective trion oscillator strength: as more and more trions are excited in the system, the extra injected photons have less electrons and trions to couple to and to form trion-polaritons. As a result the collapse of strong photon–trion coupling occurs at the density of excited trions equal to half the density of available free electrons, *n*_T_ ≈ *n*_e_/2. The results of the theoretical modelling (see ‘Methods’) are shown by the red solid curves in Fig. 3b, c reproducing the experimental *red* shift of *E*_MPB_ and the corresponding reduction of *ℏ*Ω_T_. Overall, the trion Rabi-splitting can be approximated as Ω_T_(*n*_T_) ≈ Ω_T_(0)(1 − 2*n*_T_/*n*_e_), and the value of theoretical nonlinearity is given by ${\beta }_{{\rm{T}}}^{{\rm{eff}}}=2\hslash {\Omega }_{{\rm{T}}}(0)/{n}_{{\rm{e}}}\approx 30\,\upmu {\rm{eV}}\cdot \upmu {{\rm{m}}}^{2}$ for *n*_e_ ≈ 400 μm^−2^ and *ℏ*Ω_T_(0) ≈ 5.8 meV (see Supplementary Note 3.5) in agreement with the experimental value of 37 μeV ⋅ μm^2^. It is the low electron density and high oscillator strength per single trion (large *ℏ*Ω_T_(0)), that lead to the high value of trion-polariton nonlinearity.

### Nonlinear neutral exciton–polaritons

**Fig. 4:**
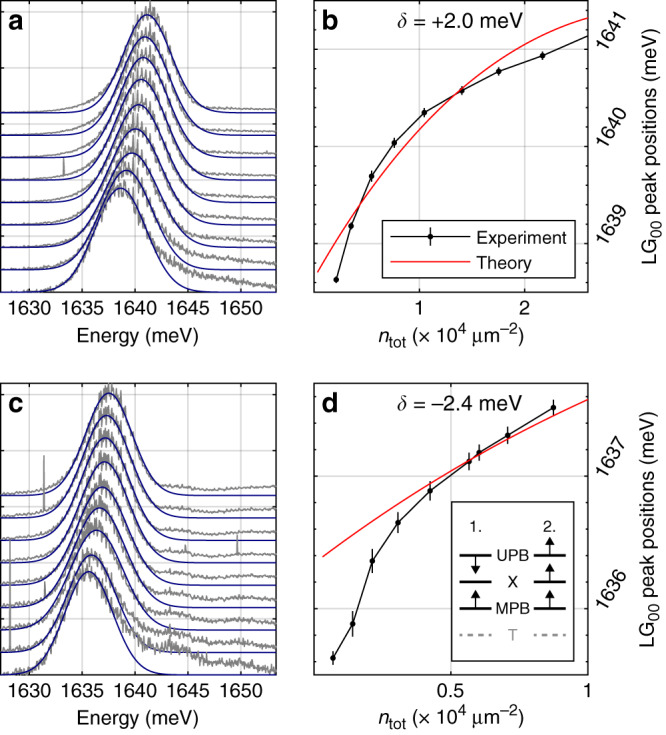
Neutral exciton–polariton nonlinearity. **a**, **b** Small positive detuning *δ*_C–X_ = +2.0 meV is considered. **a** Spectra of the MPB *LG*_00_ mode for the pump powers from 1 to 9 μW (power increases from bottom to top). **b** Extracted peak positions of the mode vs. exciton density. **c**, **d** Small negative detuning *δ*_C–X_ = −2.4 meV is considered. **c** Spectra of the MPB *LG*_00_ mode for the pump powers from 1 to 9 μW (power increases from bottom to top). **d** Extracted peak positions of the mode vs. estimated exciton density. The error bars (95% CI) in (**b**–**d**) are deduced from the fitting procedure in (**a**–**c**). Red solid curves correspond to the theoretical modelling results. Inset: schematic diagrams showing how the MPB and UPB shift in the case of phase space filling (Mechanism 1 leading to reduction of Rabi-splitting between neutral exciton level and the bare photon mode) and the neutral exciton blueshift (Mechanism 2).

Next we study the neutral exciton–polariton nonlinearity, which may arise from: (1) the reduction of exciton–photon Rabi-splitting *ℏ*Ω_X_, and/or (2) blueshift of the neutral exciton level *E*_X_^28^. Both mechanisms should lead to blueshift of the MPB. The blueshift of the MPB peak associated with the neutral exciton–polariton nonlinearity is observed in Fig. 3b for *δ*_C–X_ = −15.4 meV at *n*_tot_ > 5 × 10^2^ μm^−2^ above the threshold of strong trion–photon coupling collapse. We further studied neutral exciton–polariton nonlinearity for several photon–exciton detunings in the range from  +8.8 meV to  −2.4 meV, where the trion fraction is negligible (~3% or less; see Table II in Supplementary Information). The central laser frequency was shifted to be in resonance with the MPB in each case. Substantial blueshifts of MPB mode of the order of 2 meV are observed at much higher excitation power in the range from 1 to 9 μW (see Fig. 4a, c). The MPB energy depends sublinearly on the total exciton/trion density *n*_tot_ as shown in Fig. 4b, d. In this power range the density of excited neutral excitons *n*_X_ is much higher than that of trions and *n*_tot_ is dominated mostly by neutral excitons (*n*_tot_ ≫ *n*_e_, *n*_tot_ ≈ *n*_X_).

**Fig. 5:**
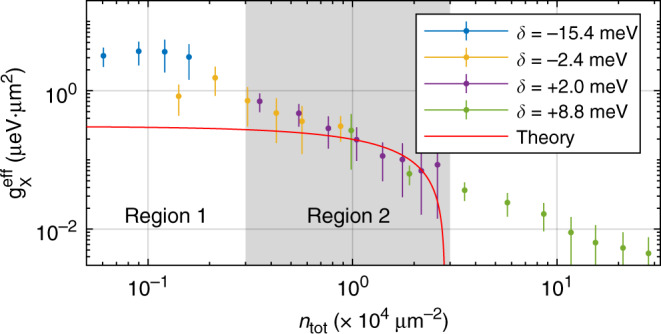
Strength of nonlinearity vs pump. The experimental effective interaction constant ${g}_{{\rm{X}}}^{{\rm{eff}}}$ is shown as a function of the estimated exciton density, *n*_tot_. The ${g}_{{\rm{X}}}^{{\rm{eff}}}$ is deduced from the energy blueshift of MPB (see Supplementary Note 1.5). The data correspond to four different cavity–exciton detunings (*δ*):  +8.8 meV (olive),  +2.0 meV (purple),  −2.4 meV (orange), and  −15.4 meV (blue). The error bars (95% CI) are deduced taking into account errors in determining the MPB peak positions at each pump power (exciton density). The red solid curve corresponds to the theoretical values.

As we discuss in Supplementary Note 1.5 at intermediate densities the optical nonlinearity arises mainly from the exciton blueshift (Mechanism 2)^28^, which is characterised by the parameter ${g}_{{\rm{X}}}^{{\rm{eff}}}=d{E}_{{\rm{X}}}/d{n}_{{\rm{tot}}}$. The ${g}_{{\rm{X}}}^{{\rm{eff}}}$ is expected to be constant in a system where only pair exciton–exciton interactions are important^28^. By contrast, Fig. 5 shows that the experimental ${g}_{{\rm{X}}}^{{\rm{eff}}}$ decreases with *n*_tot_ from ≃ 2–5 μeV ⋅ μm^2^ at *n*_to_ ~ 10^3^ μm^−2^ to  ≃0.01 μeV ⋅ μm^2^ at *n*_tot_ ~ 10^5^ μm^−2^, which suggests the importance of higher order exciton–exciton interactions. The lower values ≃ 0.05 *μ*eV ⋅ μm^2^ are similar to the values reported in WS_2_ waveguide structures, where only very high excitation powers were used^29^.

To describe the observed neutral exciton-nonlinearity we developed a model taking into account the two- and three-exciton exchange processes^30,31^ (see ‘Methods’ and Supplementary Note 2). At 3 × 10^3^ < *n*_tot_ < 3 × 10^4^ μm^−2^ within an experimental error there is agreement between theory and experiment (Fig. 5, region 2). The values of ${g}_{{\rm{X}}}^{{\rm{eff}}}$ in exciton density region 2 are also in agreement with the values of the exciton–exciton interaction parameter in monolayer WSe_2_ characterising excitation induced exciton broadening in the same density range^32^. The theory also qualitatively reproduces the sublinear shifts in Fig. 4b and d. At *n*_tot_ > 3 × 10^4^ μm^−2^ our theory is not applicable anymore, since in this case higher order nonlinearities should be taken into account. By contrast, at *n*_tot_ < 3 × 10^3^ μm^−2^ the model accounting only for exciton–exciton interactions results in the very weak theoretical blueshift of *E*_MPB_, much smaller than that observed in the experiment in Fig. 3b. This is also reflected in Fig. 5, where at *n*_tot_ < 3 × 10^3^ μm^−2^ (region 1) the theoretical ${g}_{{\rm{X}}}^{{\rm{th}}} \sim 0.3\,\upmu {\rm{eV}}\cdot \upmu {{\rm{m}}}^{2}$ is observed to be below the corresponding experimental values ${g}_{{\rm{X}}}^{{\rm{eff}}} \sim 0.5-3\,\upmu {\rm{eV}} \cdot \upmu {{\rm{m}}}^{2}$. Such a discrepancy indicates that at these exciton densities the trion-mediated exciton–exciton interactions, which are characterised by increased scattering cross-section and number of exchange processes, might play an important role in the observed large polariton blueshift (see Supplementary Note 3.6).

## Discussion

In this work we measure the nonlinearity due to trion-polaritons with respect to that due to neutral exciton–polaritons. We observed that depending on the exciton–photon energy detuning the trion-polariton nonlinearity is 10–100 times larger than that due to neutral exciton–polaritons. For example, the trion-polariton energy redshift of the order of 1 meV is observed at excitation power of  ~20 nW (see Fig. 3a) at *δ*_C–X_ = −15 meV, whereas in the case of neutral exciton–polaritons the same energy shift is observed at excitation powers of  ~400 nW and  ~4 μW at *δ*_C–X_ = −15 meV and *δ*_C–X_ = 2 meV in Figs. 3b and 4, respectively. This is consistent with fact that the deduced average value of ${\beta }_{{\rm{T}}}^{{\rm{eff}}}\simeq 37\,\upmu {\rm{eV}}\cdot \upmu {{\rm{m}}}^{2}$ is 10–100 times larger than the values ${g}_{{\rm{X}}}^{{\rm{eff}}}$ observed in Fig. 5 at *n*_tot_ < 10^4^ μm^−2^.

Determination of the measured absolute values ${\beta }_{{\rm{T}}}^{{\rm{eff}}}$ and ${g}_{{\rm{X}}}^{{\rm{eff}}}$ requires careful deduction of the total polariton (exciton/trion) density excited with a single resonant pulse in the system in order to minimise possible systematic errors. The most accurate way to do this would be to use the incident energy of the excitation pulse and the coupling efficiency of the external radiation to the 0D polariton mode. In our experiment, this coupling efficiency is not known, since the excitation beam does not match well the spatial profile of the polariton mode. Instead, we deduce the total exciton/trion density using the transmitted power and the ratio of the measured polariton linewidth to that of the bare cavity mode linewidth. The latter gives an upper bound on the ratio of the polaritons absorbed into the reservoir to polaritons emitted into free space since scattering into the reservoir cannot be faster than the total loss rate of polaritons (see discussion in Supplemental Material for more details). The accuracy of this method can be judged by the fact the values of ${g}_{{\rm{X}}}^{{\rm{eff}}}$ of about 1–0.3 μeV μm^2^ at exciton densities in the range 10^3^–10^4^ μm^−2^ derived in the current manuscript are consistent within a factor of 2–3 with ${g}_{{\rm{X}}}^{{\rm{eff}}} \sim 1\pm 0.4 \, \upmu {\rm{eV}}\cdot \upmu {{\rm{m}}}^{2}$ measured in ref. ^33^ for neutral exciton–polaritons realised in a slab waveguide photonic crystal in the same density range. In ref. ^33^ the exciton density was derived accurately using femtosecond excitation and detection and measurement of the absolute power of the resonant excitation pulse and the coupling efficiency of the incident radiation to the polariton mode. In that case polariton absorption is not relevant. Since in the current work the trion-polariton nonlinearity is measured with respect to the neutral exciton–polariton nonlinearity we can take ${g}_{{\rm{X}}}^{{\rm{eff}}}$ as a reference point and confirm that the value ${\beta }_{{\rm{T}}}^{{\rm{eff}}}\simeq 37\upmu {\rm{eV}}\cdot \upmu {{\rm{m}}}^{2}$ is likely to be accurate within a factor of two to three as well. Good agreement between our theory of the trion-polariton nonlinearity (which does not use fitting parameters) and the experiment (Fig. 3c) further supports this statement.

Notably, the deduced trion-polariton nonlinearity is of the same order as that observed in microcavities with a single quantum dot^2,3^, where strong renormalisation of Rabi-splitting (~100 μeV) occurs at a single photon level^2^. The average value of ${\beta }_{{\rm{T}}}^{{\rm{eff}}}\simeq 37\upmu {\rm{eV}}\cdot \upmu {{\rm{m}}}^{2}$ is also  ~5–10 times larger^8,28^ or comparable^9,10^ to the reported coefficients characterising neutral exciton–polariton nonlinearity in GaAs system. In terms of future perspectives the value of ${\beta }_{{\rm{T}}}^{{\rm{eff}}}$ can be further verified by studying the second-order correlation function of the emission from the resonantly driven polariton mode^13,14^. Strong interactions can lead to polariton blockade and antibunching ^13^. As we elaborate theoretically in Supplementary Note 4 polariton quantum effects can potentially be realised in high-Q microcavities with embedded high quality homogeneous TMDC samples^34^, where very narrow polariton resonances with a linewidth given just by the cavity mode lifetime could be achieved.

It is also useful to relate parameter ${\beta }_{{\rm{T}}}^{{\rm{eff}}}$ to nonlinear refractive index coefficient *n*_2_ widely used in the field of nonlinear optics to characterise Kerr-like nonlinearity of materials. We estimated the value of *n*_2_ per single flake associated with strong trion–photon coupling to be around  ~10^−10^ m^2^  ⋅ W^−1^ (see Supplementary Note 1.7). This value is 3-5 orders of magnitude greater than nonlinear refractive coefficient of 2D TMDC materials and graphene studied in the weak light–matter coupling regime (see Supplementary Note 1.7). The nonlinear refractive index *n*_2_ due to trion polaritons of a hybrid monolayer-cavity system is also found to be 3-4 orders magnitude larger than *n*_2_ coefficient of widely used bulk optical materials (e.g., Si, AlGaAs, etc.) [see Supplementary Note 1.7].

In conclusion, we observed strong Kerr-like nonlinearity associated with trion-polaritons in a TMDC system due to phase space filling of trion states. The ratio of trion- to neutral exciton–polariton interaction strength is found to be in the range from 10 to 100. The variation of neutral-exciton–polariton nonlinearity with density is attributed to higher order exciton–exciton and trion–exciton interactions. Our work paves the way towards development of *scalable* active nanophotonic devices based on 2D materials (where the exciton level is the same across large areas in contrast to 0D quantum dots) utilising the polariton nonlinearity for control of light by light, potentially at quantum level^35^.

## Methods

### Experimental technique

In our experiment a MoSe_2_ monolayer is covered with a monolayer of hexagonal boron nitride (hBN) to protect MoSe_2_ from contamination. The heterostructure of hBN/MoSe_2_ was fabricated using standard mechanical exfoliation technique and dry transfer methods. To form a microcavity the hBN/MoSe_2_ structure was positioned on top of a flat distributed Bragg reflector (DBR) (bottom mirror) consisting of 13 pairs SiO_2_/Ta_2_O_5_ quarter-wave layers. The top mirror is a 13-pair DBR deposited on a hemispherical surface, which is fabricated using focused ion beam milling. The two mirrors were then aligned and brought into a close proximity to each other (the distance between the mirrors is set to ~1 μm) using piezo nano-positioners forming an “open-cavity” system (see Fig. 1c^26^) with the resulting formation of discrete microcavity Laguerre–Gauss (LG) photonic modes. The open cavity system was positioned into bath cryostat at temperature of 4 K. For resonant excitation we employed 100 fs laser pulses with repetition rate 1 kHz. These were obtained from the frequency-doubled output of an optical parametric amplifier (Light-Conversion TOPAS) pumped by the 800 nm pulses from a Ti:Sapphire regenerative amplifier system (Spectra Physics Spitfire seeded by Spectra-Physics Mai-Tai Ti:Sapphire Oscillator). The excitation beam was focused into the spot size of about 5–10 μm on the bottom flat mirror, so that only a fraction of the incident photons couples to the highly confined *LG*_00_ cavity mode.

### Theory methods

To describe the observed polariton nonlinearity we begin with the conceptually more simple case of a neutral exciton–polariton, where the trion fraction is negligible (Fig. 4). Aiming to describe the influence of the exciton density (*n*_X_) increase, we note that the Rabi frequency Ω_X_ corresponds to the low intensity value Ω_X_(0) only for the case when excitons are ideal bosons described by a creation operator ${\hat{X}}_{{\bf{k}}}^{\dagger }$. The deviation of statistics coming from the Pauli principle leads to their changed commutation relations, $[{\hat{X}}_{{\bf{k}}},{\hat{X}}_{{\bf{k}}^{\prime} }^{\dagger }]={\delta }_{{\bf{k}},{\bf{k}}^{\prime} }-{D}_{{\bf{k}},{\bf{k}}^{\prime} }$^36^, where ${D}_{{\bf{k}},{\bf{k}}^{\prime} }$ is a non-bosonicity parameter. In the analysis we consider density-dependent Rabi-splitting *ℏ*Ω_X_(*n*_X_) and nonlinear shift of the exciton level *E*_X_(*n*_X_) (see Supplementary Note 2 for the details). The modified coupling reads

1 $${\Omega }_{{\rm{X}}}({n}_{{\rm{X}}})={\Omega }_{{\rm{X}}}(0)\left(1-\frac{8\pi }{7}{n}_{{\rm{X}}}{a}_{{\rm{B}}}^{2}+\frac{384{\pi }^{2}}{455}{n}_{{\rm{X}}}^{2}{a}_{{\rm{B}}}^{4}\right),$$

where *a*_B_ is an exciton Bohr radius in TMDC. The nonlinear shift of the exciton energy arising from the Coulomb scattering and deviation of exciton statistics from that of ideal bosons, reads

2 $${E}_{{\rm{X}}}({n}_{{\rm{X}}})=	\,{E}_{{\rm{X}}}(0)+\frac{8}{\pi }\frac{{e}^{2}}{4\pi {\epsilon }_{0}\kappa {a}_{{\rm{B}}}}{{\mathcal{I}}}_{4}({r}_{0}){n}_{{\rm{X}}}{a}_{{\rm{B}}}^{2}\\ 	-\frac{128}{5}\frac{{e}^{2}}{4\pi {\epsilon }_{0}\kappa {a}_{{\rm{B}}}}\left[\right.5{{\mathcal{I}}}_{6}({r}_{0})-2{{\mathcal{I}}}_{4}({r}_{0})\left]\right.{n}_{{\rm{X}}}^{2}{a}_{{\rm{B}}}^{4},$$

where *e* is an electron charge, *ϵ*_0_ is vacuum permittivity, *κ* denotes average dielectric permittivity, *r*_0_ is a screening parameter, and ${{\mathcal{I}}}_{4,6}({r}_{0})$ are dimensionless integrals. The linear (∝ *n*_X_) and quadratic terms ($\propto {n}_{{\rm{X}}}^{2}$) in Equations (1) and (2) take into account the two- and three-exciton exchange processes^30,31^, respectively.

We apply the above theory to explain the nonlinear MPB blueshift when trion contribution can be omitted. To do so, the exciton–photon Hamiltonian is diagonalised accounting both for *E*_X_(*n*_X_) and Ω_X_(*n*_X_), and energy for the lower polariton branch is considered^28^. The theoretical results are shown in Fig. 4b, d by the red solid curves. Performing the variational procedure to calculate the properties of excitons and matching the binding energy to experimental value, we set the exciton Bohr radius to *a*_B_ = 0.85 nm^37^ and qualitatively describe the sublinear dependence of polariton blueshift (see Supplementary Note 2). By differentiating Eqs. (1) and (2) over *n*_X_ we can obtain the corresponding theoretical ${\beta }_{{\rm{X}}}^{{\rm{th}}}$ and ${g}_{{\rm{X}}}^{{\rm{th}}}$ parameters, which characterize the neutral exciton–polariton nonlinearity due to reduction of exciton–polariton Rabi-splitting and the exciton level blueshift, respectively (see Supplementary Note 1.5).

Gaining the knowledge from the exciton case, we consider the trion-dominated regime (Figs. 2 and 3). The excitation process then corresponds to the creation of a trion from a free electron^24,38,39^. We account for phase space filling effects which become important at increasing excited trion density (see Supplementary Note 3). This leads to the quenching of the collective trion oscillator strength. Taking into account the deviation of statistics for trions from that of ideal fermions^40^ we calculate the influence of phase space filling on the trion Rabi frequency Ω_T_(*n*_T_, *λ*_1,2_) as a function of density and variational parameters for the trion wavefunction *λ*_1,2_. To describe Fig. 3b,c we perform the diagonalisation of the full photon–trion-exciton system with nonlinear contributions to both trion and exciton modes. We use *λ*_1_ = 0.86 nm, *λ*_2_  = 2.54 nm, obtained by the variational procedure, in line with computational trion studies in MoSe_2_^41^. The results are shown by the red solid curves in Fig. 3b, c reproducing the experimental red shift of *E*_MPB_ and reduction of Ω_T_.

## Supplementary information

Supplementary Information

## Data Availability

Data supporting this study are openly available from the University of Sheffield repository: 10.15131/shef.data.12523217.

## Code availability

All the detailed theoretical derivations are presented in the Supplementary materials and can be easily implemented in, for example, Mathematica package.
